# An Overview of Sport Participation and Exercise Prescription in Mitral Valve Disease

**DOI:** 10.3390/jcdd10070304

**Published:** 2023-07-18

**Authors:** Francesco Perone, Mariangela Peruzzi, Edoardo Conte, Luigi Sciarra, Giacomo Frati, Elena Cavarretta, Annachiara Pingitore

**Affiliations:** 1 Cardiac Rehabilitation Unit, Rehabilitation Clinic “Villa delle Magnolie”, Castel Morrone, 81020 Caserta, Italy; francescoperone1988@gmail.com; 2 Department of Clinical Internal, Anesthesiology and Cardiovascular Sciences, Sapienza University of Rome, 00161 Rome, Italy; mariangela.peruzzi@uniroma1.it; 3 Mediterranea Cardiocentro, 80122 Napoli, Italy; 4 Division of Cardiology, IRCCS Galeazzi Sant’Ambrogio Hospital, University of Milan, 20157 Milan, Italy; edoardo.conte86@gmail.com; 5 Department of Clinical Medicine, Public Health, Life and Environmental Sciences, University of L’Aquila, 67100 Coppito, Italy; luigi.sciarra@univaq.it; 6 Department of Medical-Surgical Sciences and Biotechnologies, Sapienza University of Rome, Corso Della Repubblica 79, 04100 Latina, Italy; giacomo.frati@uniroma1.it; 7 IRCCS Neuromed, Pozzilli, 86077 Isernia, Italy; 8 Department of General and Specialistic Surgery “Paride Stefanini”, Sapienza University of Rome, 00161 Rome, Italy; annachiara.pingitore@uniroma1.it

**Keywords:** mitral valve, athletes, mitral valve prolapse, exercise prescription, pre-participation screening, echocardiography, mitral regurgitation, mitral stenosis

## Abstract

The incidence of heart valve disease (HVD) has been rising over the last few decades, mainly due to the increasing average age of the general population, and mitral valve (MV) disease is the second most prevalent HVD after calcific aortic stenosis, but MV disease is a heterogeneous group of different pathophysiological diseases. It is widely proven that regular physical activity reduces all-cause mortality rates, and exercise prescription is part of the medical recommendations for patients affected by cardiovascular diseases. However, changes in hemodynamic balance during physical exercise (including the increase in heart rate, preload, or afterload) could favor the progression of the MV disease and potentially trigger major cardiac events. In young patients with HVD, it is therefore important to define criteria for allowing competitive sport or exercise prescription, balancing the positive effects as well as the potential risks. This review focuses on mitral valve disease pathophysiology, diagnosis, risk stratification, exercise prescription, and competitive sport participation selection, and offers an overview of the principal mitral valve diseases with the aim of encouraging physicians to embody exercise in their daily practice when appropriate.

## 1. Introduction

Regular physical activity is essential for the physical and mental wellness of all individuals and is widely proven to reduce the all-cause mortality rate [1,2,3,4,5]. This is particularly crucial in patients with cardiovascular (CV) affections or increased CV risk, in which exercise prescription also represents part of the non-pharmacological therapy. Recent evidence demonstrated that even patients affected by hypertrophic cardiomyopathy (HCM) who practiced physical activity exhibited lower CV mortality if compared with sedentary HCM patients [6]; moreover, the practice of mild to moderate exercise has proved safe and beneficial effects, despite the traditional concern about the increased risk of sudden cardiac death (SCD). The incidence of heart valve disease (HVD) has been rising over the last few decades [3], mainly due to age-related degenerative processes as one of the main causes of HVD [7]. For those patients, regular exercise is necessary to prevent physical decay and frailty. On the other side, asymptomatic or mildly symptomatic younger patients affected by degenerative mitral valve (MV) prolapse and regurgitation wish to practice high-intensity workouts or competitive sports. During intense physical activity, the increase in heart rate, preload, or afterload might worsen symptoms, accelerate progression of HVD, and potentially trigger major arrhythmias and SCD [8]. To best balance the beneficial effects of physical activity with the exercise-induced possible risks, it is essential to correctly individuate the valve defect pathophysiology, quantify the hemodynamic severity, and assess the individual arrhythmic risk before allowing or denying the competitive sport and customizing the exercise prescription [9]. More specifically, exercise prescription refers to targeted training programs that are planned by a sports cardiologist or rehabilitation specialist and are dedicated to specific patients who need to reach precise fitness goals. The exercise prescription should include indications about the training type, intensity, time, and frequency (FITT model), and it is advisable to reassess it periodically by monitoring the patient’s fitness status. Exercise types comprise mainly aerobic, resistance, or a combination of both trainings; the frequency is the number of exercise sessions per week, while the time might refer to the hours of exercise or to the duration of the exercise program in terms of weeks or months. The intensity represents the training workload and is expressed in kcal/min or metabolic equivalents (METs). The prescribed exercise intensity may be represented by a percentage of maximal aerobic capacity (VO_2_ max) evaluated at the cardiopulmonary exercise test (CPET), a percentage of the measured during effort or predicted (220-age) maximal heart rate (HRmax), or a percentage of the HR reserve (HRR) evaluated at the CPET or derived with the Karvonen formula [10]. Other alternatives for monitoring the exercise intensity are the use of “Borg’s rating of perceived exertion” or the “talk test”, which provide an estimated rate of perceived exertion, or the 6-min walking test (6 MWT), which is an indicator of submaximal exercise capacity [11]. In addition, the use of telerehabilitation programs could be reasonable in patients for whom close monitoring is not always possible, especially if the health conditions are precarious or the fitness status is compromised, which would require a continuous re-adaptation of the exercise prescription [12]. However, CPET should be the exam of choice for personalizing the exercise prescription in HVD patients since it is able to identify the aerobic capacity (VO_2_/WR) and the anaerobic threshold (analysis of VE/VO_2_ slop, the nadir of EqO_2_ and the nadir of PetO_2_), quantify the exercise capacity (VO_2_ peak, RER), identify the causes of exercise limitation, and determine variables that affect patients’ prognosis (VE/VCO_2_, VO_2_ peak) [13,14]. Moreover, the CPET allows the evaluation of other CV parameters, such as the electrocardiogram (ECG) trace, the blood pressure response, and the presence of symptoms. In this narrative/umbrella review, we appraise an overview of the principal MV disease with the aim of encouraging physicians to embody regular exercise as part of therapeutic programs by providing a practical guidance from the different MV disease pathophysiologies, exercise prescription, and competitive sport participation, as appropriate. In addition, we wish to highlight the several gaps in knowledge in this field and stimulate further research on the topic to strengthen the evidence.

## 2. Mitral Stenosis

### 2.1. Pathophysiology

Rheumatic heart disease and the degenerative calcification of the MV apparatus are the leading causes of MV stenosis. The pathophysiological mechanism of stenosis development and progression in rheumatic heart disease recognizes several mechanisms: commissural fusion, leaflet thickening, shortening and fusion of the subvalvular apparatus, and superimposed calcifications [15]. On the contrary, in cases of degenerative calcification of the MV, the calcium deposition starts from the mitral annulus (MAC), especially posteriorly, and the basal portion of the mitral leaflets, limiting their physiological excursion [16]. Another relevant cause of MV stenosis in young individuals is radiotherapy-induced HVD, which might manifest even after treatment [17]. Independently from the causes, the increase in cardiac output and heart rate in MV stenosis patients during exercise imply unfavorable hemodynamic changes. Those changes include the increase in mean gradients and filling pressures, resulting in augmented pulmonary capillary and artery pressures, favoring the onset of atrial fibrillation and/or other supraventricular tachyarrhythmias or, in the worst cases, the risk of pulmonary oedema [18]. Therefore, exercise tolerance is often limited in these patients due to the early occurrence of symptoms such as fatigue and dyspnea [18].

### 2.2. Diagnosis and Risk Stratification

Transthoracic echocardiography is extremely useful to assess the valve anatomy and the stenosis severity by combining the anatomical MV orifice assessment with the haemodynamic parameters in terms of orifice area, mean pressure gradient, and systolic pulmonary artery pressure (sPAP) estimate, which both have prognostic value [19]. In patients affected by rheumatic heart disease, echocardiography is also useful to report the disease extension/severity, which should be graded with dedicated scores with the aim of early recognizing individuals who are eligible for interventional procedures [20]. The European Guidelines [19] define a valve area between 1.5 cm^2^ and 1 cm^2^ as moderate stenosis, while a valve area <1 cm^2^ is classified as severe. Since a normal MV is about 4–6 cm^2^, an area ≤1.5 cm^2^ is already considered hemodynamically significant, even though not necessarily severe. Indeed, in moderate to severe MV stenosis, a near-normal cardiac output at rest with an impaired increase during exercise is present. Conversely, MV area >1.5 cm^2^ is defined as mild stenosis, and subjects generally remain asymptomatic (Table 1).

Risk stratification of patients with MV stenosis engaging in regular physical activity is based on clinical examination, ECG, transthoracic echocardiography, and exercise stress tests. This assessment is aimed at testing functional capacity, symptoms, and the hemodynamic impact at rest and under exertion [9]. A maximal exercise stress test with 12-lead ECG recording is performed to assess the symptoms, functional capacity, blood pressure response, inducible arrhythmias, and myocardial ischemia and to prescribe the appropriate exercise intensity. Furthermore, a CPET, which is the exam of choice when available, also provides information about respiratory gas exchange. Moreover, exercise stress echocardiography is indicated in cases of discrepancy between clinical symptoms and MV stenosis severity at rest in order to unmask a possible worsening of the functional parameters during exercise [22]. Indeed, in the case of hemodynamically significant mitral stenosis, it is essential to promptly identify symptoms such as atrial fibrillation and pulmonary hypertension, which are the leading parameters to indicate the need for surgical intervention.

### 2.3. Physical Activity and Exercise Prescription

In the literature, specific exercise programs in patients affected by mitral stenosis are lacking. Therefore, since symptomatic patients are candidates for valve interventions, it frequently happens that only asymptomatic and stable individuals are considered for exercise training. However, it might be reasonable that even symptomatic subjects be engaged in tailored training programs, which should be similar to the heart failure exercise programs, with the aim of avoiding deconditioning and the complications of a sedentary lifestyle. In patients with MV stenosis, physical activity may cause a sudden occurrence or significant worsening of symptoms, mainly due to the increased heart rate and left ventricular (LV) filling pressures, especially in case of atrial fibrillation, hence the administration of beta-blockers for an optimal rate control is crucial in view of appropriate training sessions [23].

Individuals with MV stenosis can participate in recreational sports and leisure-time exercise, according to the latest European Sports Cardiology Guidelines [10]. More specifically, subjects with mild MV stenosis can participate in all recreational and leisure-time sports in the presence of sinus rhythm, a near-normal resting systolic pulmonary artery pressure (sPAP < 40 mmHg), and normal exercise testing. Asymptomatic individuals with the same criteria but moderate MV stenosis may participate in all recreational and leisure-time sports at a low to moderate intensity. Participation in recreational exercise and leisure activity at moderate or high intensity is not recommended in patients affected by severe MVS or in symptomatic subjects; however, participation in low-intensity leisure exercises may be considered in mildly symptomatic individuals after a careful evaluation, including exercise stress echocardiography.

### 2.4. Competitive Sport Participation

**Figure 1:**
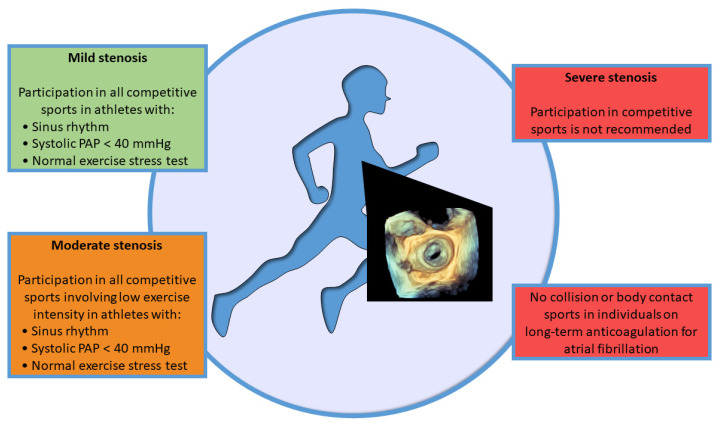
Eligibility for sport participation in asymptomatic individuals with mitral stenosis. PAP, pulmonary artery pressure.

Recommendations for participation in competitive sports in individuals with asymptomatic MV stenosis share similarities with indications for recreational/leisure-time exercises, according to the latest European Guidelines [10] (Figure 1). Indeed, subjects with mild MV stenosis in sinus rhythm, normal sPAP, and normal exercise testing can participate in all competitive sports [10]. On the other hand, subjects with moderate MV stenosis with the same characteristics may participate in low-intensity competitive sports [10]. Individuals with severe MV stenosis are advised against all competitive sports because of the high risk of complications. In addition, subjects on oral anticoagulation therapy for atrial fibrillation are not eligible for collision or body-contact sports [10].

The Italian Cardiological Guidelines for Competitive Sport Eligibility (COCIS) [24,25] are in general more restrictive than the European Guidelines [10] because only competitive sport eligibility is considered, not leisure-time physical activity. Indeed, individuals with moderate to severe MV stenosis or affected by atrial fibrillation cannot participate in any competitive sport, while selected individuals with mild or moderate MV stenosis in sinus rhythm may participate in skill sports. More specifically, should be considered eligible only patients with documented normal exercise capacity; mean transvalvular pressure gradient <15 mmHg and sPAP < 60 mmHg during exercise stress echocardiography; absence of complex arrhythmias both at the exercise stress test and at the 24-h ECG monitoring, including a specific training session [24,25].

## 3. Degenerative Mitral Regurgitation

### 3.1. Pathophysiology

Degenerative MV regurgitation (MVR) is a primary regurgitation resulting from abnormalities of the leaflets, annulus, and subvalvular apparatus, which show excessive movement [26]. MV prolapse (MVP) is defined as a systolic superior displacement of one or both leaflets >2 mm above the mitral annulus, measured using echocardiography in the long axis view. The main causes are fibroelastic deficiency and Barlow’s disease. Specifically, fibroelastic deficiency is characterized by thin leaflets and a focal lesion involving mainly the scallop P2, while Barlow’s disease presents a multi-scallop prolapse of thickened, distended, and redundant leaflets with diffuse excessive tissue; MVP is a continuum among those 2 entities, which may be represented even in the same patient’s valve [19]. Both conditions in their natural history may be complicated by the occurrence of chordal rupture, which usually implies a sudden worsening of MVR, but only bileaflet MVR due to Barlow’s disease may hold the high-risk feature of malignant mitral valve prolapse (MVP).

Exercise in patients with MVR causes hemodynamic changes based on the possible dynamic nature of valve severity, left ventricle contractile response to workload, and increased sPAP. A high heart rate or blood pressure could increase MVR and filling pressures during effort [27]. In addition, exercise induces an increase in LV ejection fraction due to the reduction in systolic volume [22]. However, despite normal LV systolic function at rest, exercise could unmask left ventricle dysfunction, resulting in impaired contractile reserve. Furthermore, individuals with MVR during exercise could develop right ventricular dysfunction in addition to exercise-induced pulmonary hypertension [28,29].

### 3.2. Diagnosis and Risk Stratification

Transthoracic echocardiography provides details on the anatomy of the MV complex, the mechanism causing regurgitation, and the hemodynamic consequences on cardiac chambers and pulmonary pressure. Evaluation of MVR severity is based on multi-parameter assessment, including the integration of qualitative, semi-quantitative, quantitative, and structural parameters. Qualitative parameters are MV morphology, color flow jet, flow convergence zone, and continuous wave signal of the jet. Semi-quantitative parameters are vena contracta (VC) width, pulmonary vein flow, mitral inflow, and the mitral-to-aortic flow-velocity integral ratio. Quantitative parameters are effective regurgitant orifice area (EROA), regurgitant volume (RVol), and regurgitant fraction. Instead, structural parameters are LV and left atrium (LA) size and pulmonary pressures. Recommended measurements for grading regurgitation are the VC width and the proximal isovelocity surface area (PISA) method, while the other parameters are useful for confirming the severity [30]. Furthermore, LV size, LV ejection fraction, and sPAP guide risk assessment and exercise prescription [10], Table 2.

Risk stratification is based on clinical and physical examination, ECG, echocardiography, and the maximal exercise stress test [9]. Clinical evaluation is based on investigating symptomatic status and functional capacity. An exercise stress test evaluates the presence of arrhythmias during exercise as well as symptoms, ischemic threshold, and hemodynamic response. The protocol test should be based on the type of sport and its expected intensity level. In addition, exercise echocardiography is useful in cases of discrepancy between symptoms and MVR severity, to stratify risk, and to identify predictors of adverse outcomes. Indeed, markers of a worse prognosis tested during exercise are dynamic MVR with increased severity, sPAP ≥ 60 mmHg, failure to increase LV ejection fraction, and induced right ventricular dysfunction. Other parameters with negative impacts are poor functional capacity, the occurrence during exertion of major ventricular arrhythmias (VAs) or atrial fibrillation, and failure to recover heart rate [22].

### 3.3. Arrhythmogenic Mitral Valve Prolapse and Mitral Annulus Disjunction

MVP is generally a benign condition with a reported prevalence of 1–3% [31,32]. However, the incidence of SCD related to MVP appears to be about 2–4% [33,34], and the SCD related to MVP resulted in 7% in young adults of an Italian registry, with a major prevalence in young females [35]. As already mentioned, the MVP is represented by a wide variety of anatomical presentations, but only bileaflet Barlow’s disease has been associated with SCD, in addition to other high-risk features such as female sex, family history of MVP or SCD, personal history of syncope or pre-syncope, polymorphic VAs or right bundle branch block (RBBB) and superior axis morphology VAs at rest and during efforts, presence of mitral annulus disjunction (MAD) and of LV fibrosis at CV magnetic resonance (CMR) with gadolinium [36,37]. MAD is defined as the mitral annulus detachment from the basal LV myocardium; it occurs more often posteriorly since the anterior leaflet is in continuity with the fibrous trigone of the cardiac skeleton. Some authors recognize the existence of a MAD as an abnormal insertion of the hinge line of the posterior mitral leaflet on the atrial wall and a pseudo-MAD [38]. The presence of MAD has been documented in about 33% of patients affected by MVP [39]. It has been described that MAD is a “marker” of arrhythmic MVP and myocardial fibrosis, with a direct correlation between MAD severity and ventricular arrhythmic burden [40], but there is not universal consensus on the pathological significance of isolated MAD.

The arrhythmogenesis is secondary to the coexistence of a substrate, such as myocardial fibrosis, the modulating factors, namely the increased adrenergic tone during exercise, and the triggers [41]. More specifically, the mechanical tension induced by the billowing leaflet favors the stretching and fibrosis of the structure supporting the MV apparatus, the basal inferolateral myocardium, and the papillary muscles [35]. Concurrently, the myocardial traction alters the ventricular action potential and impairs the distal Purkinje fibers function [42,43]. Finally, an increased sympathetic tone has been identified in patients with MVP [44], which further increases during efforts and exercise, in particular during competitions due to the emotional involvement.

In this scenario, before considering a sport prescription, a cautious assessment of the arrhythmogenic risk is essential to identify patients at elevated risk of SCD or complex Vas, regardless of the degree of MVR. Several authors suggest collecting family and personal histories, to perform a physical examination, resting and stress 12-lead ECG, transthoracic echocardiography, and 24-h ECG motoring as baseline examinations [37,45,46,47]. When the ECG monitoring or the clinical information raises concern for arrhythmic events, a CMR or even an electrophysiological study might be indicated [48]. MVP can be present in familiar clusters; therefore, an echocardiographic screening of close relatives is recommended [49]. Among familiar forms, an X-linked transmission related to the FLNA gene and an autosomal dominant mode involving MVPP genes, including DCHS1 and DZIP1, have been reported to be involved in both familiar and isolated forms [50,51].

### 3.4. Physical Activity and Exercise Prescription

Asymptomatic individuals with mild MVR can participate in all recreational and leisure-time sports, as well as competitive sports. Instead, asymptomatic patients with moderate MVR could participate only in low- or moderate-intensity recreational sports, such as skill sports, in selected cases with preserved LV ejection fraction (≥60%), LV end-diastolic diameter <60 mm (or <35.3 mm/m^2^ in men and <40 mm/m^2^ in women), sPAP < 50 mmHg at rest, and a normal maximal exercise stress test [10]. According to the same criteria, asymptomatic individuals with severe MVR may practice low-intensity prescribed exercise and should be evaluated for early reparative MV surgery in a high-volume center to guarantee MV repair [19]. Finally, subjects with symptomatic MVR and reduced exercise capacity should not participate in any sport, although low-intensity supervised aerobic exercise should be recommended to improve physical capacity before cardiac surgery (pre-habilitation) [52]. In women affected by MVP, a 12-week aerobic training program (3 times a week, heart rate between 60% and 85% of maximum, intensity levels increasing over time) improved symptoms, functional capacity, and well-being and reduced anxiety, atypical chest pain, and fatigue related to MVP syndrome [53].

### 3.5. Competitive Sport Participation

Asymptomatic individuals with/without MVP and mild MVR can participate in all competitive sports with annual echocardiographic monitoring. If high-risk features for arrhythmogenic MVP are present, a case-by-case evaluation by a referral sports cardiology center is needed [9,10,37,54].

**Figure 2:**
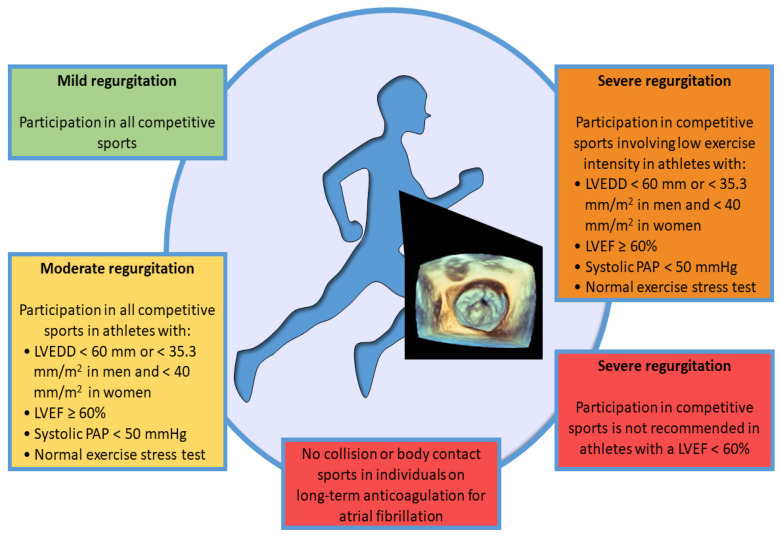
Eligibility for sport participation in asymptomatic individuals with degenerative mitral regurgitation.

Individuals with severe MVR cannot participate in competitive sports if their LV ejection fraction <60% as this indicates severe LV systolic dysfunction and should be immediately referred for eventual cardiac surgery. In addition, even asymptomatic individuals with severe MVR cannot participate in competitive sports if they involve moderate or severe exercise intensity. Instead, in the presence of specific criteria and in sports with low exercise intensity, asymptomatic subjects with severe MVR may participate in competitive sports. Specifically, these individuals may participate if left ventricle function is preserved (LV ejection fraction ≥60%) with left ventricular end-diastolic diameter <60 mm (or <35.3 mm/m^2^ in men and <40 mm/m^2^ in women), resting sPAP is <50 mmHg, and an exercise stress test documents a good functional capacity and the absence of hemodynamic disturbances and complex arrhythmias [9,10]. Furthermore, asymptomatic individuals with the same criteria should participate in all competitive sports with a moderate MVR. Individuals anticoagulated for atrial fibrillation should avoid contact or collision sports (Figure 2).

As with mitral stenosis, the Italian Guidelines for Competitive Sport Eligibility (COCIS) [24,25], are more restrictive than the European Guidelines for individuals with MVR. Indeed, individuals with severe MVR cannot participate in any competitive sports. Athletes with moderate MVR can engage in skill sports and may participate in moderate and high-dynamic competitive sports in selected cases. Specifically, these individuals may be considered eligible for 6 months under close monitoring of left ventricle size and contractile function at rest and during effort, evaluated with exercise echocardiography.

## 4. Secondary Mitral Regurgitation

### 4.1. Pathophysiology

Secondary MVR, also known as functional MVR, occurs on structurally normal MV leaflets and chords and is due to leaflet tethering, mostly caused by LV dysfunction and mitral annular enlargement, often linked to LA dysfunction, and then sustained by longstanding LA volume overload itself [55]. The LV remodeling may be due to coronary heart disease or non-ischemic cardiomyopathy. In both cases, the wall motion abnormalities and/or the LV dilation, most of the time with papillary muscle displacement/dysfunction, result in a reduction of LV closing forces and increased leaflet tethering forces [56]. Nevertheless, a severe LA dilation, usually associated with permanent atrial fibrillation, can cause an atrial functional MVR [55] due to mitral annular dilation with the impairment of leaflet coaptation. The ischemic MVR, particularly related to anterior myocardial infarction, also involves a mitral annulus deformation that appears dilated and flattened with a loss of its physiological saddle shape [57]. Moreover, another functional MVR contributor is the LV mechanical dyssynchrony induced by the possible coexistence of intra-ventricular conduction delays, which could worsen with exercise if rate-dependent [58,59]. Characteristically, functional MVR changes its degree of severity as a function of hemodynamic conditions and rest or exercise situations. In the vast majority of patients affected by MVR secondary to LV dysfunction, regardless of the cause, both the volume and the pressure loading tend to increase, respectively, with aerobic or isometric exercise, without modifications in LV ejection fraction [60,61,62,63]. Indeed, even if the elevated blood pressure values during exercise and the consequent rise in afterload favor an increase in the LV and mitral closing forces, the tethering forces are still prevailing, resulting in a worsening of MVR severity [63]. This condition may be further aggravated by the coexistence of LV dyssynchrony and mitral annular dilation, which worsen with exercise [64,65]. The LV volumes appear to be greater in ischemic cardiomyopathy, and the exercise-induced deterioration of MR depends on the LV scar extent and localization [65]. Therefore, the main predictors of exercise-induced MVR worsening are mainly the LV sphericity and the MV tenting parameters, rather than LV volumes or ejection fraction [60]. Moreover, the deterioration of MVR during exercise is inversely related to the LV contractile reserve and does not depend on MVR severity at rest [60]. In patients with significant LA enlargement, even when the LV is normal, the lonely mitral annulus dilatation is sufficient to compromise the coaptation surface and so favor the MVR [55,66].

### 4.2. Diagnosis and Risk Stratification

The quantification of MV disease requires both qualitative and quantitative echocardiographic measurements, similarly to primary MVR. Qualitative measurements include: MV morphology, color flow, and continuous wave Doppler evaluation of the regurgitant jet. Characteristically, the ischemic MVR jets are central in the case of leaflet symmetric tethering and/or annular dilation, or rather posteriorly directed when there is posterior leaflet tethering prevailing [67,68]. The quantitative assessment consists of flow convergence analysis that, by measuring the PISA, allows to derive the EROA, RVol, and regurgitant fraction. However, in ischemic MVR, it often happens that the regurgitant orifice exhibits an elliptical shape or that the orifice appears greater in the early systole than in the mid systole due to its dynamic nature; hence, the grading of ischemic MVR severity may e misleading. In these cases, or when multiple regurgitant orifices are present, the 2D-3D non-invasive hemodynamic evaluation of the RVol might be more reliable [67,68]. Finally, the semi-quantitative methods include the VC width, but this measurement as well tends to underestimate the MR degree because of the EROA ovoidal shape [69]. In the latest ESC guidelines on HVD, the ischemic MVR severity parameters differ from primary MVR only for LV and left atrium structural abnormalities, which are not graded in ischemic MVR, and for EROA and RVol, which are allowed to be lower in case of elliptical regurgitant orifice area or low flow conditions [19]. However, additional parameters (i.e., the coaptation depth, the tenting height and area, the mitral annulus diameters, and the VC area) are needed in ischemic MVR, especially to better understand the mechanism of the regurgitation and to guide the interventional/surgical therapies. In this regard, a comprehensive 2D-3D echocardiography evaluation of the MV apparatus and of the mitral annulus is often crucial [26,70].

The secondary MR risk stratification largely depends on the underlying cardiac disease and is based on clinical and physical examination, laboratory tests (i.e., BNP or NTproBNP in heart failure), ECG, echocardiography, maximal exercise stress test/CPET, cardiac magnetic resonance, and 24-h ECG monitoring. Indeed, the aim of risk stratification is to define the arrhythmic risk, assess the functional capacity, point out symptoms or hemodynamic instabilities, and prescribe exercise intensity on the basis of maximal exercise capacity and heart rate response. However, the risk stratification of patients affected by ischemic MVR, especially in cases of coronary artery disease and heart failure, should always include the markers of exercise-induced adverse prognosis, thus classifying individuals into low-risk and high-risk groups. Independently from the underlying condition, the presence at rest of ischemic MVR represents a marker of a more severe disease, especially in patients affected by heart failure, and an estimated EROA of >20 mm^2^ represents an indicator in increased mortality rate [71]. Moreover, the worsening of ischemic MVR degree with exercise is related to impaired exercise tolerance and a worse prognosis because of the reduced stroke volume and the increase of pulmonary and LA pressures [72,73,74]. Therefore, in the risk stratification flowchart, stress echocardiography should be considered to unmask the dynamic component of secondary MR. More specifically, a rise in the mortality rate has been reported in patients with heart failure who showed an increase in EROA > 13 mm^2^ during exercise [60]. Many authors have observed that patients affected by ischemic MR, especially in the presence of myocardial scars, exhibit a worse prognosis with respect to MVR due to a non-ischemic cardiomyopathy [64,68,75]. This is due to multiple reasons, such as the underlying coronary artery disease with possible incomplete revascularization or gradual disease progression, the increased risk of fatal arrhythmias, and the self-maintaining/worsening of function of mitral regurgitation, especially in cases of severe myocardial fibrosis [68,76]. Conversely, patients with atrial MVR exhibit better outcomes, although they often present high filling pressures [66]. In cases of atrial fibrillation, the maximal workload or Borg’s rating of perceived exertion are the only parameters to guide an exercise test or stress echocardiography.

### 4.3. Physical Activity and Exercise Prescription

Cardiac rehabilitation effectively represents a milestone in CV disease therapy due to the positive effect of regular exercise on the significant reduction of mortality and CV event rates and the improvement of exercise capacity [1,2,4,5]. On the other hand, intense training might favor life-threatening arrhythmias in this population; hence, to avoid the risk of SCD, it is essential to individualize the exercise prescription that should follow the FITT (frequency, intensity, time in terms of duration, and type of exercise) model. In this regard, exercise prescription in patients with functional MR depends greatly on the underlying disease.

#### 4.3.1. Coronary Artery Disease

In the case of coronary artery disease, it is crucial to identify the possible presence of high-risk features for exercise-induced adverse cardiac events (i.e., critical coronary stenosis, reduced LV ejection fraction, inducible myocardial ischemia, recent revascularization/acute coronary syndrome, Vas), which should guide the exercise prescription on an individual basis [10]. Equally important is considering the type and level of the practiced sport and the patient’s fitness level. Subjects that complete a normal exercise or functional imaging test can return to sport 3–6 months after coronary revascularization [10]. Meanwhile, patients affected by untreatable myocardial ischemia might practice low-to-moderate recreational exercise under clinical surveillance and leisure sports (2–3 times/week) while maintaining the exercise intensity under (c.a. 10 beats) the ischemic and arrhythmic threshold [10,76].

#### 4.3.2. Heart Failure

In patients affected by heart failure, exercise-based cardiac rehabilitation should be reserved for all clinically stable subjects receiving optimal medical therapy under strict clinical observation (exercise prescription tailoring every 3–6 months and anytime the intensity of exercise is increased) with the aim of improving exercise tolerance, quality of life, and reducing hospital readmissions [3,5]. Endurance exercises (at least 3–5 days/week; 20–60 min per session) with a gradual increase of the sport intensity up to 85% VO_2_peak or to the maximum workload tolerated are always recommended [3]; the inclusion of high-intensity interval training (HIIT) might be considered for low-risk patients [77,78,79]. Complementarily, resistance training (2–3 day/week with an intensity of Borg’s rating of perceived exertion <15) resulted to be safely combined with aerobic training in order to increase skeletal muscle mass and quality of life [80]. Moreover, even respiratory exercises targeted to improve the cardiopulmonary status, especially in compromised patients, are part of the correct training prescription [81].

### 4.4. Competitive Sport Participation

In sports cardiology, European guidelines on the indication for competitive sports in cases of secondary MVR are subject to the underlying disease. In cases of coronary artery disease, individuals with no inducible ischemia and arrhythmias, a normal LV ejection fraction, and no symptoms can be considered for competitive sports on an individual basis, with some restrictions for power, mixed sports, and endurance activity in patients older than 60 years [10]. On the contrary, coronary angiography should be performed in patients with documented inducible ischemia, although optimal medical therapy and high-risk lesions should be treated. Thereafter, if a normal provocative test is performed about 3–6 months after the revascularization is performed, these patients might return to being engaged even in intensive exercise programs. Competitive sports are restricted to individuals with documented inducible ischemia and/or coronary lesions, which cannot be treated effectively with medical therapy nor with revascularization; only skill sports with low-intensity training can be considered [10]. In patients affected by heart failure, which underpins the secondary MR, before being considered for sport participation, it is important to exclude contraindications to exercise, optimize the medical and device therapy, and perform a complete clinical and functional evaluation. Although non-competitive sports, even at moderate to high intensity, may be considered for selected individuals with mid-range ejection fraction, participation in competitive sports cannot be advised by both European and Italian guidelines [10,25]. The latter [25] specifies that individuals with severe secondary forms of MVR should not engage in any competitive sports. However, after mitral intervention (mitral valve repair or Mitra-Clip repair), the indications may be reconsidered through an accurate global assessment including echocardiography, a stress test, and 24-h ECG monitoring. Moreover, according to the latest update [25], subjects affected by coronary artery disease, which is often complicated by secondary MVR, who are considered at low risk may be eligible for skill sports only. However, the exception exists for individuals with dilated cardiomyopathy, who are deemed at low risk, or carriers of implantable cardioverter-defibrillators (ICD), to get involved in competitive skill sports with low CV demand.

## 5. Percutaneous and Surgical Mitral Valve Repair and Replacement

### 5.1. Percutaneous Mitral Valve Interventions

Transcatheter edge-to-edge repair (TEER) is the technique with the greatest evidence in patients affected by degenerative MVR with high risk for surgery or in secondary MVR patients who are not eligible for surgery and who do not need coronary revascularization [19]. Specifically, the improvement of the functional class and the decrease of rehospitalizations and mortality rates are renowned, mainly in functional MVR patients [19].

Patients with functional MVR who underwent TEER showed an improvement in CPET parameters, including the peak VO_2_, likely due to the increase in anterograde stroke volume, decrease in pulmonary pressures, and reverse remodelling of the LV [81,82]. On the contrary, it has been reported that individuals affected by degenerative or secondary MVR treated with TEER did not exhibit an increase in peak VO_2_ despite the improvement in cardiac output [83]. The authors have interpreted these results as the expression of a reduced arterial-venous O_2_ content difference at the peak of exercise with respect to the pre-operative assessment.

However, there are no dedicated studies on the outcome of rehabilitation after the TEER procedure nor precise guidelines on exercise prescription or competitive sport participation in this specific category of patients. Therefore, it might be reasonable to apply the risk stratification and exercise prescription advice suggested for heart valve diseases and/or heart failure, depending on the percutaneous intervention result and on the presence of comorbidities.

### 5.2. Surgical Mitral Valve Repair or Replacement

Surgical treatment of MV disease includes prosthetic valve replacement (with a mechanical or biological prosthesis) or native valve repair. MV repair is the first-choice intervention in case of degenerative MVR, with an expected durable result [19]. This type of surgery represents the best alternative therapy to valve replacement in degenerative MVR due to the preservation of the native valve apparatus, lack of anticoagulation three months after the intervention, and excellent hemodynamic performance [19,84]. The reparability of segmental valve prolapse is associated with a low risk of reoperation, while extensive prolapse, rheumatic alterations, and extensive valve calcifications reduce the feasibility of intervention. In the case of MV replacement, the choice of prosthesis is based on several parameters, such as age, life expectancy, patient preference, risks with long-term anticoagulation, and comorbidities that may condition future re-intervention.

Patients with normal-functioning prosthetic MV have a minimal increase in gradient during exercise. However, hemodynamic response to physical stress could be suboptimal, with a negative impact on performance, despite normal values at rest [22,54]. There is limited data on the natural history of valve replacements or repairs in patients who exercise intensely. Individuals with prosthesis dysfunction, such as stenosis or prosthesis-patient mismatch, show reduced exercise capacity and an associated significant increase in gradients and pulmonary hypertension [9]. Furthermore, functional stenosis with reduced exercise capacity could occur in patients after MV annuloplasty [22]. Finally, after MV replacement, careful monitoring should be performed to assess the possible development of tricuspid regurgitation and right ventricular dysfunction with related symptoms [85].

### 5.3. Physical Activity, Exercise Prescription, and Competitive Sport Participation

It has been demonstrated that early cardiac rehabilitation after valve surgery is effective in improving exercise and functional capacity [86] and significantly decreasing the mortality rate [87,88,89]; moreover, these beneficial effects appear to last over time [87]. Nevertheless, a recent systematic review of randomized controlled trials enrolling patients after heart valve surgery or percutaneous procedures showed controversial results [90].

**Figure 3:**
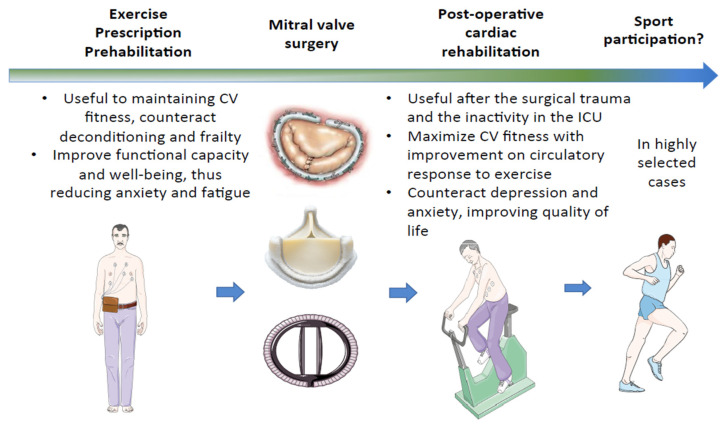
Prehabilitation, cardiac rehabilitation, exercise prescription and competitive sport eligibility after mitral valve surgery. CV, cardiovascular; ICU, intensive care unit.

A recent position statement of the Sport Cardiology Section of the European Association of Preventive Cardiology [9] advises that individuals who underwent MV replacement should receive the same exercise prescription as those with asymptomatic moderate native MV disease if the LV function is preserved and the sPAP normal. In contrast, in patients with mechanical prostheses or atrial fibrillation, since anticoagulation is mandatory, the opportunities for physical activity and competitive sports participation are naturally limited. Indeed, in this patient category, the risks of physical trauma during sports should always be considered, and high-impact contact physical activities are strongly discouraged. On the other hand, eligibility for competitive sports after MV repair is related to the residual degree of valve stenosis or regurgitation [10]. Moreover, physical activity following MV surgery can be performed 3 months after surgery, but only in cases of normal LV function and preserved exercise capacity [9]. Cardiac rehabilitation is suggested during the 12 weeks after cardiac surgery to gradually improve the CV response to exercise, functional capacity, and quality of life (Figure 3). Indeed, the post-operative period is usually characterized by reduced CV fitness due to the physical stress induced by surgery, anemia, and inactivity during intensive care.

Exercise prescription following MV repair or prosthetic valve replacement is based on clinical assessment, ECG, echocardiography, and exercise testing. The exercise stress test is performed to best evaluate the type, intensity, duration, and type of sport (FITT model) to be engaged. However, a CPET assessment with 12-lead ECG recording should be performed, if available. In subjects who underwent surgical mitral repair, eligibility can be reconsidered on the basis of the disease evolution, the repaired valve functionality (mild or zero residual MR) at rest and during effort, the LA and LV size, and the absence of VAs at basal and stress ECG and at 24-h ECG monitoring, including a training session. Individuals with prosthetic valves or valve repair should undergo annual re-evaluation with the stress test or CPET, defying the exercise intensity to pursue [9,54].

The Italian Guidelines for Competitive Sport Eligibility [25] consider athletes with biological or mechanical prostheses eligible for skill sports in the case of a normally functioning prosthesis both at rest and during effort (exercise stress echocardiography), preserved ventricular function, and the absence of major arrhythmias at the 24-h ECG monitoring that includes a specific training session. In selected cases, suitability for low-to moderate intensity sports with annual revision may be considered by ensuring accurate monitoring over time of LV function and transvalvular gradient during exercise (increase in mean exertional gradient <10 mmHg for mitral valve prostheses).

## 6. Discussion and Future Perspectives

Population studies report that the prevalence of significant MV regurgitation, both primary and secondary, is the second most frequent valve defect after aortic valve stenosis, followed by aortic regurgitation and mitral stenosis [3]. Besides the medical therapy and conventional recommendations reserved for patients affected by HVD, such as antibiotic prophylaxis and scrupulous oral hygiene for the prevention of endocarditis [91,92], regular physical activity should represent a building block of therapeutic interventions, independent of the patients’ age or disease severity.

Position papers and guidelines [9,10,25,54] are present in the literature, but systematic research on physical activity, exercise prescription, and competitive sport participation in all types and severity of MV diseases is missing. Our review seeks to fill this gap by suggesting a practical approach to the management of sport practice when each MV disease occurs.

Although the CV benefits of physical activity are fully proven, there is still much to be accomplished to make it a cornerstone of global wellness for patients with MV diseases, regardless of age or comorbidities. Indeed, physicians are frequently more prone to consider the importance of exercise in young individuals or in cases of non-severe MV defects, whereas it is sometimes undermined in older aged or more severely affected subjects (i.e., heart failure, coronary heart disease), and it is common that patients with acute onset of diseases are discharged without clear indication on when and how to re-engage in physical activity.

However, precise sport prescription and follow-up timing should be tailored by trained physicians to the type and severity of the defect and to the health conditions of the patient, with the aim of maintaining a good fitness status and avoiding major cardiac events [93]. In this regard, accurate assessment of the patients’ comorbidities and multi-parametric cardio-vascular evaluations are preparatory to safe and individualized training guidance.

As a general principle, individuals affected by a mild MV disease with both stenosis and regurgitation and no high arrhythmic risk indicators are not subjected to specific restrictions in terms of exercise practice or competitive sport participation. On the contrary, in the presence of moderate to severe MV disease, high-risk patients, or concomitant comorbidities, cautious risk stratification and multiple investigations are recommended before any training prescription.

After corrective surgery, patients improve clinically and may return to engaging in regular physical activity. Further studies are necessary to assess the long-term consequences of exercise in this profile of patients. Furthermore, the impact of the hemodynamic response during physical activity should be studied for the different types of prosthetic valves. Currently, recommendations for athletes after surgical intervention are conservative due to the absence of data. Large-scale longitudinal studies are required to provide further indications on exercise prescription and sport participation. In addition to the assessment of LV function and transvalvular gradient over time, other parameters should also be evaluated and monitored, such as LA function and size [94]. Finally, the role of stress echocardiography should be better defined in athletes with MV disease or after corrective surgery. New studies investigating echocardiographic parameters associated with poor prognosis and high-risk events during different types of exercise, sports, and intensities should be conducted. Advanced echocardiography, such as deforming imaging, could better stratify athletes and their responses to exercise. Stress echocardiography could be useful in the surveillance of individuals with native valve disease to assess the impact of physical activity on valve disease progression. Furthermore, this method could add useful information on the natural history of patients with valve replacement or repair.

## 7. Conclusions

Regular physical activity is a key therapy for individuals with CV diseases. Benefits are multiple, but potential exercise-induced risks of adverse events and complications must be evaluated. In individuals with MV disease or after corrective surgery, careful assessment and risk stratification are necessary to evaluate the appropriate exercise prescription and participation in competitive sports.

## Figures and Tables

**Table 1:** Mitral valve stenosis grading criteria based on rest and stress 2D echocardiography. MS, mitral stenosis; PAP, pulmonary artery pressure.

	Mild	Moderate	Severe	Variation during Stress Echocardiography
Valve Area (cm^2^)	>1.5	1.0–1.5	<1.0	--
Mean transmitral pressure gradient (mmHg)	<5	5–10	>10	MS is severe if mean gradient increases: >15 mmHg at peak physical exercise >18 mmHg during dobutamine infusion
Systolic Pulmonary Artery Pressure (mmHg)	<30	30–50	>50	A systolic PAP elevation >60 mmHg or a rapid increase in systolic PAP (>90% increase at second step of exercise) are highly predictive of exercise-induced dyspnea

Adapted from [15,21].

**Table 2:** Mitral valve regurgitation grading criteria based on rest and stress 2D echocardiography. EROA, effective regurgitant orifice area; LA, left atrium; LV, left ventricle; LVOT, left ventricular outflow tract; MR, mitral regurgitation; MV, mitral valve; PA, pulmonary arterial; PH, pulmonary hypertension; PISA, proximal isovelocity surface area; sPAP, systolic pulmonary artery pressure; TVI = time-velocity integral; VC, vena contracta.

	Mild	Mild-to-Moderate	Moderate-to-Severe	Severe		Variation during Stress Echocardiography
Qualitative Parameters				Primary MR	Secondary MR	
MV morphology	None-mild leaflets abnormalities/tenting	Moderate leaflets abnormalities/tenting	Moderate leaflets abnormalities/tenting	Flail leaflet, ruptured papillary muscle, severe retraction, large perforation	Normal leaflets with severe tenting and/or poor leaflet coaptation	
Color Flow MR jet	Small, central	Intermediate	Intermediate	Large central jet or eccentric jet reaching the posterior wall of the LA	Large central jet or eccentric wall impinging jet of variable size	
Flow convergence zoneContinuous wave signalof MR jet	No or small faint/parabolic	Dense, partial or parabolic	Dense, parabolic or triangular	Large throughout systole Holosystolic/dense/triangular	Large throughout systole Holosystolic/dense/triangular	
**Semi-quantitative parameters**						
VC width (mm)	<3	3–<5	5–<7	≥7	≥7	
Pulmonary vein flow	Systolic dominance	Normal or systolic blunting	Systolic blunting	Systolic flow reversal	Systolic flow reversal	
Mitral inflow	A wave dominant	Variable	Peak E wave >1.2 m/s	Peak E wave >1.2 m/s	Peak E wave >1.2 m/s	
TVI mitral/TVI LVOT	<1	Intermediate	>1.2	>1.4	>1.4	
**Quantitative parameters**						
EROA (2D PISA, mm^2^)	<20	20–29	30–39	≥40	≥40 (may be ≥30 if elliptical regurgitant orifice area)	Increase in EROA ≥ 13 mm^2^ is associated with poor prognosis in secondary MR
Regurgitant volume (mL)	<30	30–44	45–59	≥60	≥60 (may be ≥45 mL if low flow conditions)	
Regurgitant fraction (%)	<30	30–39	40–49	≥50	≥50	
**Structural Parameters**						
LA size	Usually Normal	Normal or dilated	Usually dilated	Dilated	Dilated	
LV size	Usually Normal	Normal or dilated	Usually dilated	Dilated	Dilated	Absence of contractile reserve is associated with poor prognosis in primary MR
PA pressures	Usually Normal	Normal or elevated	Normal or elevated	Usually elevated	Usually elevated	Dynamic PH (sPAP ≥ 60 mmHg) is associated with poor prognosis in primary and secondary MR

Adapted from references [19,22,30].

## Data Availability

Data are available upon request.
